# The Parkinson’s Disease-Linked Protein DJ-1 Associates with Cytoplasmic mRNP Granules During Stress and Neurodegeneration

**DOI:** 10.1007/s12035-018-1084-y

**Published:** 2018-04-19

**Authors:** Mariaelena Repici, Mahdieh Hassanjani, Daniel C. Maddison, Pedro Garção, Sara Cimini, Bhavini Patel, Éva M. Szegö, Kornelis R. Straatman, Kathryn S. Lilley, Tiziana Borsello, Tiago F. Outeiro, Lia Panman, Flaviano Giorgini

**Affiliations:** 1 Department of Genetics and Genome Biology, University of Leicester, Leicester, LE1 7RH UK; 2 MRC Toxicology Unit, Leicester, LE1 9HN UK; 3 Neuroscience Department, IRCCS-Istituto Di Ricerche Farmacologiche “Mario Negri”, Milan, Italy; 4 Department of Experimental Neurodegeneration, Center for Nanoscale Microscopy and Molecular Physiology of the Brain (CNMPB), Center for Biostructural Imaging of Neurodegeneration (BIN), University Medical Center Göttingen, Waldweg 33, 37073 Göttingen, Germany; 5 Centre for Core Biotechnology Services, University of Leicester, Leicester, LE1 7RH UK; 6 Cambridge Centre for Proteomics, Department of Biochemistry, University of Cambridge, Cambridge, UK; 7 Department of Pharmacological and Biomolecular Sciences, Università degli Studi di Milano, Milan, Italy; 8 Max Planck Institute for Experimental Medicine, Göttingen, Germany; 9 Institute of Neuroscience, The Medical School, Newcastle University, Framlington Place, Newcastle Upon Tyne, NE2 4HH UK

**Keywords:** Parkinson’s disease, DJ-1, Stress granules, RNA-binding proteins

## Abstract

Mutations in the gene encoding DJ-1 are associated with autosomal recessive forms of Parkinson’s disease (PD). DJ-1 plays a role in protection from oxidative stress, but how it functions as an “upstream” oxidative stress sensor and whether this relates to PD is still unclear. Intriguingly, DJ-1 may act as an RNA binding protein associating with specific mRNA transcripts in the human brain. Moreover, we previously reported that the yeast DJ-1 homolog Hsp31 localizes to stress granules (SGs) after glucose starvation, suggesting a role for DJ-1 in RNA dynamics. Here, we report that DJ-1 interacts with several SG components in mammalian cells and localizes to SGs, as well as P-bodies, upon induction of either osmotic or oxidative stress. By purifying the mRNA associated with DJ-1 in mammalian cells, we detected several transcripts and found that subpopulations of these localize to SGs after stress, suggesting that DJ-1 may target specific mRNAs to mRNP granules. Notably, we find that DJ-1 associates with SGs arising from *N*-methyl-d-aspartate (NMDA) excitotoxicity in primary neurons and parkinsonism-inducing toxins in dopaminergic cell cultures. Thus, our results indicate that DJ-1 is associated with cytoplasmic RNA granules arising during stress and neurodegeneration, providing a possible link between DJ-1 and RNA dynamics which may be relevant for PD pathogenesis.

**Electronic supplementary material:**

The online version of this article (10.1007/s12035-018-1084-y) contains supplementary material, which is available to authorized users.

## Introduction

DJ-1 is encoded by *PARK7*, a gene associated with autosomal recessive forms of Parkinson’s disease (PD). Since the original study linking DJ-1 to PD [1], several DJ-1 mutations have been associated with familial forms of PD, with both homozygous and compound heterozygous mutations causing early onset PD [2]. DJ-1 is a small conserved protein of 189 residues implicated in a variety of cellular roles, including response to oxidative stress, mitochondrial health, protein chaperone activity, and regulation of autophagy [3, 4]. However, this plethora of DJ-1 functions makes it difficult to discern the key molecular mechanisms that connect DJ-1 to PD pathogenesis. One hypothesis is that there might be one as yet undiscovered overarching function that explains these roles in the cell [5]. Importantly, DJ-1 was first identified as part of an RNA-binding complex [6] and exhibits RNA-binding activity in human dopaminergic neuroblastoma cells and mouse brain [7]. More notably, the association of DJ-1 with specific mRNA transcripts has been demonstrated in human brain, alongside an alteration in their corresponding protein levels in PD brains [8]. We have recently made the observation that Hsp31, a yeast DJ-1 homolog, is localized to stress granules (SGs) and P-bodies (PBs) after glucose starvation and that its deletion influences formation of these cytoplasmic mRNP granules [9].

SGs are cytoplasmic aggregates that represent the morphological consequence of an mRNA triage process triggered by environmental stresses [10]. These structures are characterized by the presence of the translationally silent 48S preinitiation complex (mRNA transcripts, 40S ribosomal proteins, eIF3, eIF4A, eIF4B, eIF4G and eIF4E, and PABP-1) and represent the physical place within the cytoplasm of stressed cells where the fate of mRNA transcripts is decided. PBs, on the other hand, are RNA granules that mediate RNA degradation and dynamically interact with SGs [11], exchanging several components [12]. Interestingly, SGs co-localize with insoluble protein aggregates in several neurodegenerative diseases such as Alzheimer’s disease (AD), frontotemporal dementia and parkinsonism linked to chromosome 17 (FTDP17), and amyotrophic lateral sclerosis (ALS), suggesting shared mechanisms regarding RNA dynamics among these disorders [13, 14]. In several of these cases, mutations in RNA binding proteins increase their self-assembly ability, resulting in SG formation even in the absence of stress. Persistent SGs can also be the consequence of mutations in proteins involved in SG clearance [15]. In both scenarios, the accumulation of “chronic” SGs completely alters the RNA machinery and may trigger neurodegeneration [14, 16].

Here, we investigated the potential association of DJ-1 with SGs and PBs in mammalian systems and assessed the functional consequences of these interactions. Using mass spectrometry and co-immunoprecipitation (coIP), we find that SG components interact with DJ-1. We demonstrate that DJ-1 localizes to SGs and PBs after hyperosmotic shock and oxidative stress in HEK 293T and SH-SY5Y cells. In addition, we detected DJ-1-specific interactions with a subset of mRNAs that localize to SGs upon hyperosmotic shock. Notably, we also observed that DJ-1 co-localizes with SGs arising from neurodegeneration in primary cortical neurons and embryonic stem cell-derived dopaminergic neuronal cultures.

## Materials and Methods

### Cell Culture, Transient Transfection, and Stress Treatment

HEK 293T cells were cultured in Dulbecco’s modified Eagle’s medium (DMEM), high glucose, supplemented with 10% fetal bovine serum (FBS), 100 units/ml penicillin, and 100 μg/ml streptomycin, at 37 °C in a 95% air/5% CO^2^ atmosphere. SH-SY5Y cells were cultured in D-MEM/F12 (1:1) GlutaMAX, supplemented with 10% FBS, 100 units/ml penicillin, and 100 μg/ml streptomycin in a 95% air/5% CO^2^ atmosphere. Cells were plated on 10 cm Petri dishes (2 × 10^6^ cells/well) for immunoprecipitation studies, on coverslips (1.5 × 10^5^ cells/well) pre-coated with 0.01% poly-l-lysine solution for immunocytochemistry studies, or in 6-well plates (1.5 × 10^5^ cells/well) pre-coated with 0.01% poly-l-lysine solution for *Park7* siRNA experiments. Transfection was performed 24 h after plating using the Effectene Transfection Reagent kit (QIAGEN) using procedures supplied by the manufacturer. d-Sorbitol (Sigma) was diluted in standard growth medium to yield a 0.4 or a 0.2 M concentration. For oxidative stress treatment, 24 h after transfection, cells were exposed to 200 μM paraquat for 24 h or to 1 mM hydrogen peroxide for 2 h. Cycloheximide (CHX; Sigma-Aldrich) was used at 50 μg/ml for 30 min.

### Immunoprecipitation

To identify interaction partners of GFP-tagged DJ-1, we used the GFP-Trap technique, a high-quality GFP binding system based on a single domain antibody against GFP derived from *Camelids*. Cells were lysed 48 h after transfection. Each confluent 10 cm cell culture dish was washed twice in ice-cold phosphate buffer saline (PBS) and lysed on ice for 5 min in 400 μl lysis buffer (20 mM Tris HCl, pH 7.4, 150 mM NaCl, 1% (*v*/*v*) Triton X100 supplemented with Roche EDTA free complete mini protease inhibitors and PhosSTOP phosphatase inhibitors). Lysates were centrifuged at 14,000 rpm for 15 min at 4 °C. Supernatants were collected and GFP-trap beads (20 μl per reaction, Chromotek) were used according to the manufacturer instructions to immunoprecipitate GFP-DJ-1. Lysates from untransfected cells were used as negative controls. To identify endogenous DJ-1 interacting proteins, Dynabeads M-270 Epoxy were coated with a polyclonal goat anti-DJ-1 antibody (ab4150, Abcam) using the Dynabeads Antibody Coupling Kit (Invitrogen). Typically, 5 μg of antibody were used per 1 mg of Dynabeads M-270 Epoxy and 1.5 mg of Ab-coupled beads was used per reaction. Cell lysis was performed as described above and immunoaffinity purification was achieved by mixing at 4 °C for 1 h 30 min. Magnetic beads were then collected using a magnet and washed three times with dilution buffer (lysis buffer with no Triton). The DJ-1 protein complex was eluted from the beads for 10 min at 75 °C in 1× SDS sample buffer. DJ-1 (exogenous and endogenous) complex was separated on a 10% SDS PAGE, stained with Coomassie Blue stain compatible with mass spectrometry (ProtoBlue safe, National Diagnostic), and sent for mass spectrometry analysis at the Cambridge Centre for Proteomics, University of Cambridge, UK. For the validation of DJ-1 interaction partners, the immunocomplex was analyzed by immunoblotting. In the case of RNase A treatment, the enzyme was added to lysates to yield final concentrations of 1 mg/ml, and lysates were left at room temperature for 25 min followed by incubation with the beads as described above.

### Mass Spectrometry Analysis

Each gel lane was cut into five equally sized bands and washed, reduced in 2 mM DTT for 1 h at RT, alkylated in 10 mM Iodoacetamide for 30 min at RT and digested in-gel with 2 μg sequencing-grade porcine trypsin (Promega) overnight at 37 °C. Digests were concentrated using a speedvac and resuspended in 0.1% formic acid. All LC-MS/MS experiments were performed using a Dionex Ultimate 3000 RSLC nanoUPLC (Thermo Fisher Scientific Inc., Waltham, MA, USA) system and a QExactive Orbitrap mass spectrometer (Thermo Fisher Scientific Inc., Waltham, MA, USA). Separation of peptides was performed by reverse-phase chromatography at a flow rate of 300 nl/min and a Thermo Scientific reverse-phase nano Easy-spray column (Thermo Scientific PepMap C18, 2 μm particle size, 100 A pore size, 75 μm i.d. × 50 cm length). Peptides were loaded onto a pre-column (Thermo Scientific PepMap 100 C18, 5 μm particle size, 100 A pore size, 300 μm i.d. × 5 mm length) from the Ultimate 3000 autosampler with 0.1% formic acid for 3 min at a flow rate of 10 μl/min. After this period, the column valve was switched to allow elution of peptides from the pre-column onto the analytical column. Solvent A was water + 0.1% formic acid and solvent B was 80% acetonitrile, 20% water + 0.1% formic acid. The linear gradient employed was 2–40% B over 30 min. The LC eluant was sprayed into the mass spectrometer by means of an Easy-spray source (Thermo Fisher Scientific Inc.). All *m*/*z* values of eluting ions were measured in an Orbitrap mass analyzer, set at a resolution of 70,000. Data-dependent scans (Top 20) were employed to automatically isolate and generate fragment ions by higher energy collisional dissociation (HCD) in the quadrupole mass analyzer and measurement of the resulting fragment ions was performed in the Orbitrap analyzer, set at a resolution of 17,500. Peptide ions with charge states of 2+ and above were selected for fragmentation. Post-run, the data was processed using Protein Discoverer (version 1.4, ThermoFisher). Briefly, all MS/MS data were converted to mgf files and these files were then submitted to the Mascot search algorithm (Matrix Science, London UK) and searched against the Uniprot human database (UniProt_Human_Oct13 9606, 153,168 sequences; 54,677,058 residues) using a fixed modification of carbamidomethyl (C) and a variable modification of oxidation (M). The peptide mass tolerance was set to 10 ppm, the fragment ion mass tolerance to 0.1 Da, and the maximum number of missed cleavages to 2. Peptide identifications were accepted if they could be established at greater than 95.0% probability. emPAI scores as calculated as part of the MASCOT search algorithm (Matrix Science, London) was used for semi-quantitative analysis.

### Immunoblotting

Cells were washed twice with sterile PBS and then lysed on ice for 10 min in lysis buffer [17]. Lysates were centrifuged at 13,000 rpm for 10 min at 4 °C. Supernatants were collected and protein concentration was determined by the Bradford method. Samples were stored at − 80 °C until used. Proteins were separated on a 10% SDS polyacrylamide gel (10 μg of total proteins per well) and transferred to a polyvinylidene difluoride membrane. Membranes were incubated for 1 h in TBST 5% dried milk to saturate all non-specific binding sites. Incubation with primary antibodies was overnight at 4 °C, using mouse anti-DJ-1 antibody (1:1000; sc-55572, Santa Cruz Biotechnology), rabbit anti-β-tubulin (1:1000; #2128, Cell Signaling Technology), rabbit anti-eIF4A3 (1:1000; ab32485, Abcam), or goat anti-TIA1 (1:200; sc-1751, Santa Cruz Biotechnology). Blots were developed using horseradish peroxidase (HRP)-conjugated secondary antibodies (1:10000; Vector Laboratories) and the ECL chemiluminescence system (SuperSignal West Dura Extended Duration Substrate, Thermo Scientific).

### siRNA Knockdown of DJ-1

ON-TARGETplus human *PARK7* (11315) siRNA, SMARTpool (catalog no L-005984-00-0005) was purchased from Dharmacon siRNA Technologies (GE Healthcare) and dissolved in 1X siRNA Buffer to obtain a 20 μM stock stored in aliquots at − 20 °C before use. ON-TARGETplus non-targeting pool siRNA (catalog no D-001810-10-05) was used as a negative control, siGLO Red (catalog no D-001630-02-05) was used as transfection control, and ON-TARGETplus GAPD Control Pool (catalog no D-001830-10-05) was used as a positive control. HEK 293T cells were transfected according to the manufacturer’s specifications using DharmaFECT 1 Transfection Reagent and treated with sorbitol or lysed 72 h after transfection.

### Immunofluorescence

Cells were fixed in 4% paraformaldehyde in PBS for 20 min at 37 °C and then incubated in 1% bovine serum albumin (BSA) in PBS 0.2% Triton for 30 min at room temperature. Primary antibodies were diluted 1:100 (anti-DJ-1, #5933, Cell Signaling Technology), 1:100 (anti-DJ-1, sc-55572, Santa Cruz Biotechnology), 1:500 (anti-DJ-1, NBP1-92715, Novus Biologicals), 1:200 (anti-G3BP, #611126, BD transduction Laboratories), 1:200 (anti-eIF3η (N-20), sc-16377, Santa Cruz Biotechnology), 1:100 (anti-TIA1 (C-20), sc-1751, Santa Cruz Biotechnology), 1:1000 (anti-p54-RCK, A300-416, Bethyl Laboratories), 1:1000 (anti-p70 S6 kinase α/Hedls, sc-8418 Santa Cruz Biotechnology), 1:500 (anti-Tau 5, Calbiochem #577801), 1:200 (anti-eIF4A3, ab32485, Abcam) in blocking solution and incubated overnight at 4 °C. After washing in PBS, cells were incubated for 2 min in 1:2000 Hoechst 33342 trihydrochloride, 10 mg/ml solution (Invitrogen), in PBS. Secondary antibodies conjugated to Alexa 488, Alexa 546, Alexa 594, Alexa 647 (Invitrogen) were diluted 1:500 in PBS 0.2% Triton + 1% BSA and incubated at room temperature for 1 h. Finally, cells were rinsed in PBS, and coverslips were mounted in Mowiol.

### Confocal Laser Scanner Microscopy Analysis

Confocal laser scanner microscopy analysis (CLSM) analysis was performed using an Olympus FV1000 confocal laser scanning microscope. Cells were imaged in sequential mode using a 60X UPlanSAPO Olympus objective, and Kalman filter of 4. The following settings were used for: Hoechst—excitation 405 nm laser line, emission detected between 425 and 475 nm; Alexa 488—excitation 488 nm laser line, emission detected between 500 and 545 nm; Alexa 546—excitation 559 nm laser line, emission 575–675 nm; Alexa 594—excitation 559 nm laser line, emission 575–675; Alexa 647, emission—excitation 635 nm laser line, emission 655–755 nm. The number of SGs/PBs per cell and their average size were counted using the Stress Granule Counter plug-in (Ann Sablina, Lomonosov Moscow State University, Russia) for ImageJ software (Schneider et al. 2012) with the following parameters: number of smoothes: 10, number of smoothes after subtraction: 2, threshold: 3000, Min part size: 2, Max part size: 10, circularity: 0.2. Ten confocal z-slices taken in ten separated fields were counted in each experiment. At least 350 cells were counted per condition for each independent experiment. Cell nuclei were counted manually using the Cell Counter plug-in for ImageJ. For co-localization studies, the ImageJ co-localization plug-in written by Pierre Bourdoncle was employed. Three independent experiments were performed for all conditions.

### Precipitation of DJ-1 RNA Complexes

Immunoprecipitation reactions were as described in [18]. HEK 293T cells were allowed to reach confluence in 15 cm petri dishes (*n* = 4) and harvested in polysome lysis buffer (PLB) (10 mM Hepes, pH 7.0, 100 mM KCl, 5 mM MgCl_2_, 0.5% NP-40, 1 mM DTT, 100 units/ml RNase OUT and protease inhibitors). Each plate yielded approximately 200 μl of lysate, 100 μl of which was used in each DJ-1 and IgG control IP. DJ-1 (Abcam, ab4150) or IgG isotype control (Abcam, ab37373) antibodies were conjugated to Protein G Dynabeads (Invitrogen) for 10 min at RT with rotation. The conjugated beads were incubated with cell lysate in NET2 buffer (50 mM Tris-HCl, pH 7.4, 150 mM NaCl, 1 mM MgCl_2_, 0.05% NP-40, 20 mM EDTA, 1 mM DTT, 100 units/ml RNase OUT) for 1 h at RT and then washed six times with cold NT2 buffer.

### QPCR Analysis of Transcripts

RNA was released from the protein-bead complex by treatment with Proteinase K (beads were resuspended in NT-2 buffer supplemented with 1% SDS, 1.2 mg/ml Proteinase K) and was purified using acid phenol-chloroform followed by precipitation in 100% ethanol containing 0.27 M ammonium acetate, 0.12 M lithium chloride, and 5 mg/ml glycogen (Ambion) at − 80 °C for 16 h. Precipitated RNA was pelleted and washed with 80% ethanol before resuspension in RNase-free H_2_O. Then, 40 ng of RNA from each sample was used to synthesize cDNA with the Sensiscript® Reverse Transcription kit (QIAGEN) according to the manufacturer’s protocol.

Further, 1 μl of cDNA was used per technical replicate in a 10 μl reaction QPCR reaction with Maxima SYBR Green master mix (Thermo Scientific) and primers at a final concentration of 330 nM. QPCR reactions were performed on a LightCycler 480 system (Roche). Amplification specificity was confirmed by melt curve analysis of QPCR products and –RT controls were included for each sample. Crossing points (Cp) were calculated using the second derivative method. The ratio of mRNA levels in DJ-1 to IgG control samples was calculated using the qpcR package in R Studio [19]—amplification efficiencies were calculated using non-linear regression of sigmoidal curves and incorporated into the ratio calculation. Statistical significance of relative expression levels was tested using a pairwise-reallocation test based upon that used by REST software [20], where Cp and efficiency values were permutated within control and treatment groups. Ratios were calculated for each permutation and compared to ratios obtained from the original data. The proportion of ratios higher or lower than that obtained from the original data was used to generate the *P* value of the test.

### mRNA In Situ Hybridization

Cells were plated on NuncLab-Tek II CC2 chamber slides (ThermoFisher Scientific) and fixed with 10% NBF before being processed for RNA ISH using the RNAscope Technology, Multiplex fluorescent assay, Advanced Cell Diagnostics, Hayward, CA, USA. RNAscope probes were designed and provided by Advanced Cell Diagnostics, Hayward, CA, USA: Hs-GPX4 (NM_001039847.2, region 9-943), Hs-EIF4B (NM_001300821.1, region 472-1419), Hs-EIF4EBP1 (NM_004095.3, region 20-863). Positive and negative control probes were respectively Hs-PPIB (NM_000942.4, region 139-989) and DapB (EF191515, region 414-862). All probes were designed as C1 target probes. Staining steps were in accordance with RNAscope protocols with one modification: protease III was incubated for 5 min, as 10 min resulted in a weaker and less clear IF staining. AMP4-AltA-FL was used for the fluorescent labeling (channel 1 in green). For sorbitol-treated cells (1 h, 0.4 M sorbitol), immunofluorescence was performed following RNA ISH: after the last washes at the end of the RNAscope assay protocol, slides were washed in PBS and incubated in 1% bovine serum albumin (BSA) in PBS 0.2% Triton for 30 min at room temperature. Primary antibodies concentration was increased by 100%, as the ISH involves proteolytic treatment which might destroy the antigen of interest. Secondary antibodies were conjugated to Alexa 594 (Invitrogen) for eIF3 and TIA1, or Alexa 647 (Invitrogen) for G3BP and used as previously described. The number of ISH positive dots per cell was counted using the Stress Granule Counter plug-in as previously described for ImageJ software [21]. Ten confocal z-slices taken in ten separated fields were counted in each experiment and about 100 cells were counted per condition for each independent experiment. Cell nuclei were counted manually using the Cell Counter plug-in for ImageJ. For co-localization studies, the co-localization plug-in for ImageJ was used.

### Cortical Neuronal Culture

Primary neuronal cultures were obtained from the cortex of 2-day-old rat pups, incubated with 200 U of papain for 30 min at 34 °C and after trypsin inhibitor treatment (T-9253, Sigma Aldrich, St Louis, USA; 10 μg, 45 min at 34 °C) were mechanically dissociated. For immunocytochemistry, neurons were plated at densities of 70,000 cell/dish on chamber slides (80826, IBIDI, München, Germany) precoated with 25 μg/ml poly-d-lysine (Sigma P6407). The plating medium consisted of B27/Neurobasal-A supplemented with 0.5 mM glutamine, 100 U/ml penicillin, and 100 μg/ml streptomycin (all from Invitrogen). Experiments were performed after 12 days in culture at which time neurons had formed synapses. Cortical neurons were exposed to *N*-methyl-d-aspartate (NMDA) 100 μM for 5 or 24 h.

### Lactate Dehydrogenase Assay

Twelve DIV primary cortical neurons were treated with NMDA 100 μM for 5 or 24 h. At the end of the treatment, the medium was collected and the release of lactate dehydrogenase (LDH) in the medium was quantified to assess cell viability using the Cytotoxic 96 non-radioactive cytotoxicity assay kit (Promega, WI, USA).

### Maintenance and Differentiation of mES Cells

The NesE-Lmx1a ES cell line was propagated on MEF cells as described [22, 23]. The mES cells were differentiated following the 5-stage protocol [24] with some minor modifications. For the initiation of EB formation (stage 2), cells were dissociated with TryplE Express (Gibco) and purified on gelatinized tissue culture dishes for 45 min. Cells were subsequently plated on non-adherent bacterial dishes for 3 days in EB medium containing FBS (10%; Gibco) to allow EB formation taking place. EBs were subsequently plated on tissue culture dishes and allowed to attach. After attachment, EB medium was changed next day for DMEM/F12 (Gibco) medium containing insulin (Gibco), apo-transferrin, sodium-selenite, and fibronectin (all Sigma) (ITSF medium; stage 3). After 6 days in ITSF medium, neural precursor cells were further expanded and patterned by splitting the cells with TryplE Express and plating them on poly-ornithine and laminin (Sigma) coated 24-well plates containing N3 medium plus 10 ng/ml bFGF (R&D), 100 ng/ml FGF8 (R&D), and 100 nM Hedgehog agonist Hh-Ag1.3 (Curis Inc., USA) (stage 4). After 4 days, neuronal differentiation was initiated by removal of the growth factors and the cells were subsequently kept in N3 medium containing ascorbic acid for 12 days (stage 5). At this stage, the neuronal cultures were treated with either MPP^+^ (10 and 20 μM), rotenone (50 nM), or DMSO as control for 3, 6, and 12 h. Cell were fixed with 2% PFA for 20 min and analyzed by immunohistochemistry. The following antibodies were used: 1:500 rabbit Nurr1 (E20; Santa Cruz Biotechnology), 1:1000 mouse TH (Millipore), and 1:200 goat eIF3η (N-20, Santa Cruz Biotechnology).

### Statistical Analysis

Most data were analyzed with Prism 5 (GraphPad), using one-way ANOVA followed by post-hoc analysis with Tukey’s test. For two independent samples, an unpaired *T* test was used.

Two-way ANOVA was used for the analysis of CHX effect on SG formation followed by post-hoc analysis with Tukey’s test. *P* values of less than 0.05 were considered significant for any set of data. In all experiments, results are expressed as means ± SEM.

## Results

### DJ-1 Interacts with Stress Granule Components

To investigate the potential association of DJ-1 with SGs, we sought to identify DJ-1 interacting proteins using proteomic analyses. HEK 293T cells were transfected with a construct encoding a GFP tagged version of DJ-1 yielding expression levels comparable to those of the endogenous protein [25], and DJ-1 complexes were immunoprecipitated using the GFP-Trap technique. In parallel, we also performed coIP of proteins interacting with endogenous DJ-1. These experiments were performed in both control conditions and after oxidative stress induced by paraquat treatment (24 h, 200 μM). Purified proteins were then separated by SDS-PAGE, stained with Coomassie and each gel lane excised into equal-sized segments and in-gel digested with trypsin, before analysis of the resulting extracted peptides via liquid chromatography tandem-mass spectrometry (LC-MS/MS). Mass spectrometry identified several putative DJ-1 interactors, including SG-associated proteins (Table S1). Indeed, we found that several universal SG markers are DJ-1 interactors in both control and oxidative stress conditions (e.g., eIF4A3, a splicing factor core component of the exon junction complex (EJC) [26], and the 40S ribosomal proteins S25, S3, and S7). In addition, we identified several hnRNPs as DJ-1 interactors (hnRNPM, hnRNPA2/B1, hnRNPA1, hnRNPV, hnRNPH). Intriguingly, certain hnRNPs are known to be localized within stress granules [27–30].

Next, we assessed the relative abundance of proteins associated with DJ-1 by carrying out a semi-quantitative analysis calculating an Exponentially Modified Protein Abundance Index (emPAI) score for each protein [31]. This method calculates the number of peptides generated per protein and uses the proportion of these compared with the number of peptides which could be theoretically generated per protein as a read out of abundance. The highest emPAI scores were recorded for 40S S25 and eIF4A3 associated with endogenous DJ-1 in control conditions (emPAI score 1.04 and 1.39 respectively), with an increasing trend in levels under oxidative stress observed for both endogenous and overexpressed DJ-1. To further interrogate these data, we performed coIP experiments and found that both eIF4A3 and hnRNPM specifically interact with endogenous DJ-1 (Fig. S1A; data not shown). To gain further insight into the association of DJ-1 with SGs, we compared our mass spectrometry results to the unfixed stress granule proteome recently published by Jain et al. [32]. Notably, 33 (~ 24%) of the 139 SG proteins identified in non-fixed cells were found to be DJ-1 interacting proteins in our studies. These data indicate that DJ-1 is associated with SG-related proteins in mammalian cells, suggesting a potential novel role in RNA dynamics.

### DJ-1 Localizes to Stress Granules and P-Bodies upon Induction of Stress

**Fig. 1:**
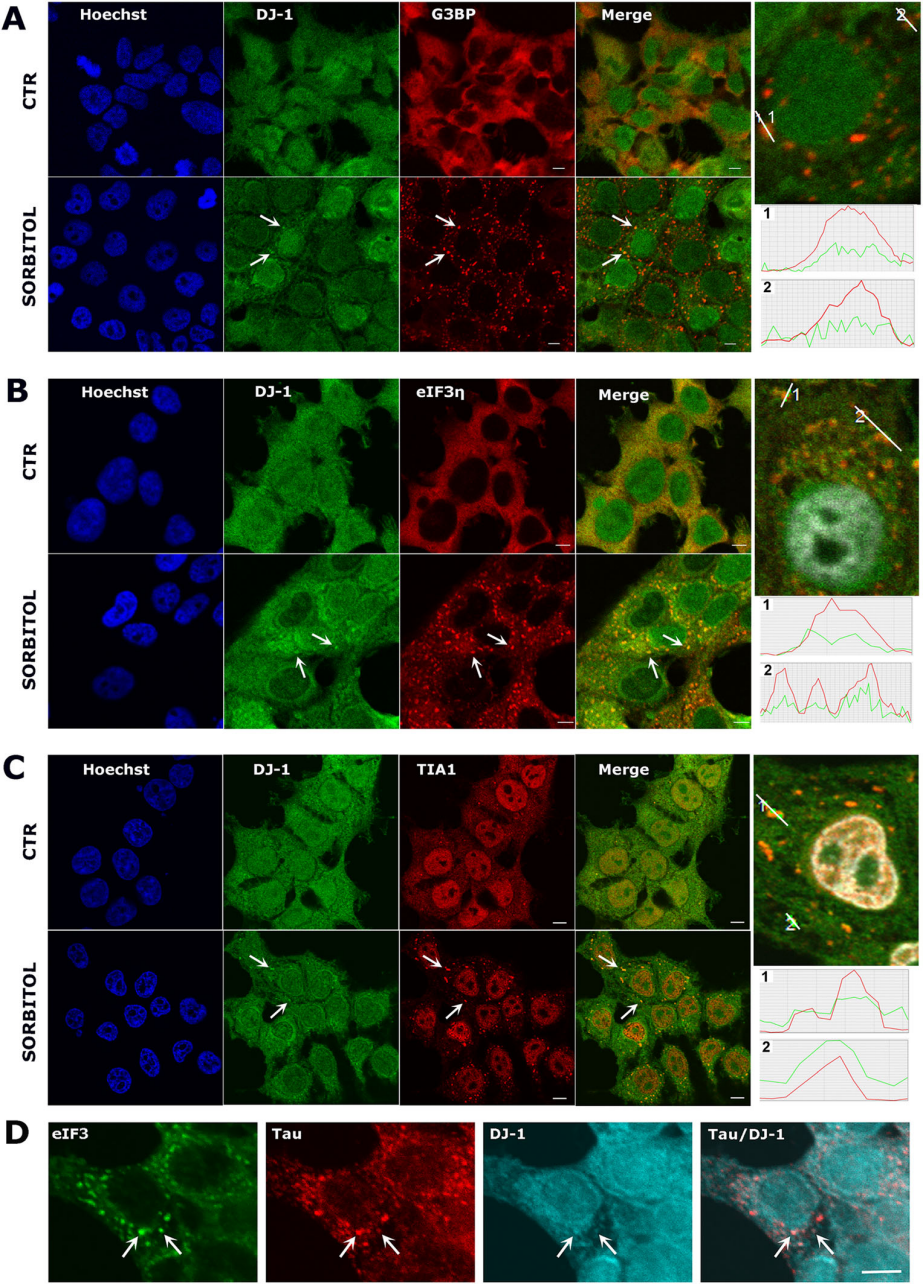
DJ-1 localizes to stress granules after hyperosmotic stress**.** Confocal images of untreated HEK 293T cells (top row in each panel) compared to cells treated with 0.4 M sorbitol for 2 h (**a**, **b**) or 1 h (**c**) (bottom row in each panel). DJ-1 changes its cellular distribution becoming more dotted and perinuclear after sorbitol treatment. Double immunostaining for DJ-1 and G3BP (**a**), DJ-1 and eIF3η (**b**), and DJ-1 and TIA1 (**c**) clearly shows DJ-1 co-localization with some stress granules, as indicated in the intensity profile data (the *y*-axis represents fluorescence intensity, the *x*-axis represents the length of the line drawn in the picture above). Images are representative of at least *N* = 3 experiments. Scale bar = 5 μm. Triple immunostaining for eIF3η, tau, and DJ-1 in SH-SY5Y cells shows DJ-1 and tau colocalization within SGs after sorbitol treatment (**d**). Scale bar = 5 μm

We then investigated the subcellular localization of DJ-1 under SG inducing conditions to assess if DJ-1 co-localizes with SGs. Sorbitol treatment inducing hyperosmotic shock was employed as a standard approach to promote formation of SGs, as determined by labeling with three established markers (G3BP, eIF3η, and TIA1). Sorbitol treatment (0.4 M) resulted in the formation of large cytoplasmic inclusions detected with G3BP (Fig. 1a), eIF3 (Fig. 1b), and TIA1 (Fig. 1c) antibodies, as previously described [33]. Interestingly, sorbitol changed the localization of DJ-1 from homogeneous nuclear and cytoplasmic distribution, in control conditions, to more perinuclear and “inclusion-like” signals after sorbitol treatment. Although there were fewer DJ-1 positive cytoplasmic inclusions than labeled SGs, there was a clear overlap in the signals, confirming the occurrence of DJ-1 in SGs, as shown in the intensity profile data (Fig. 1a–c). This subcellular localization pattern is in agreement with previous data that found a large proportion of SG components also localized to the cytoplasm during stress [34], as well as these RNA granules. By quantifying DJ-1/TIA1 co-localization, we found that ~ 55% of DJ-1 positive granules were also TIA1 positive after 1 h of treatment with 0.4 M sorbitol.

We subsequently used coIP assays to determine whether DJ-1 was physically associated with the SG markers used in the ICC studies. We immunoprecipitated endogenous DJ-1 from HEK 293T cell lysates after sorbitol treatment and compared the signal obtained to that from control beads. Neither G3BP nor eIF3η physically interacted with DJ-1 (data not shown). However, we found that DJ-1 specifically interacted with the 15 kDa TIA1 isoform, which is strongly associated with SGs, and not with the 40 kDa TIA1 isoform (Fig. S1B) [35, 36]. It has recently been shown that the protein tau—which plays a role in stabilizing microtubules and is linked to the pathology of both AD and PD—localizes to SGs and interacts with TIA1 [37, 38]. We thus analyzed tau and DJ-1 expression by ICC in SH-SY5Y cells after sorbitol treatment and we found that tau and DJ-1 co-localize within SGs identified by eIF3η (Fig. 1d), further confirming the association of these proteins to SGs, and highlighting a potentially novel pathogenic link between them.

**Fig. 2:**
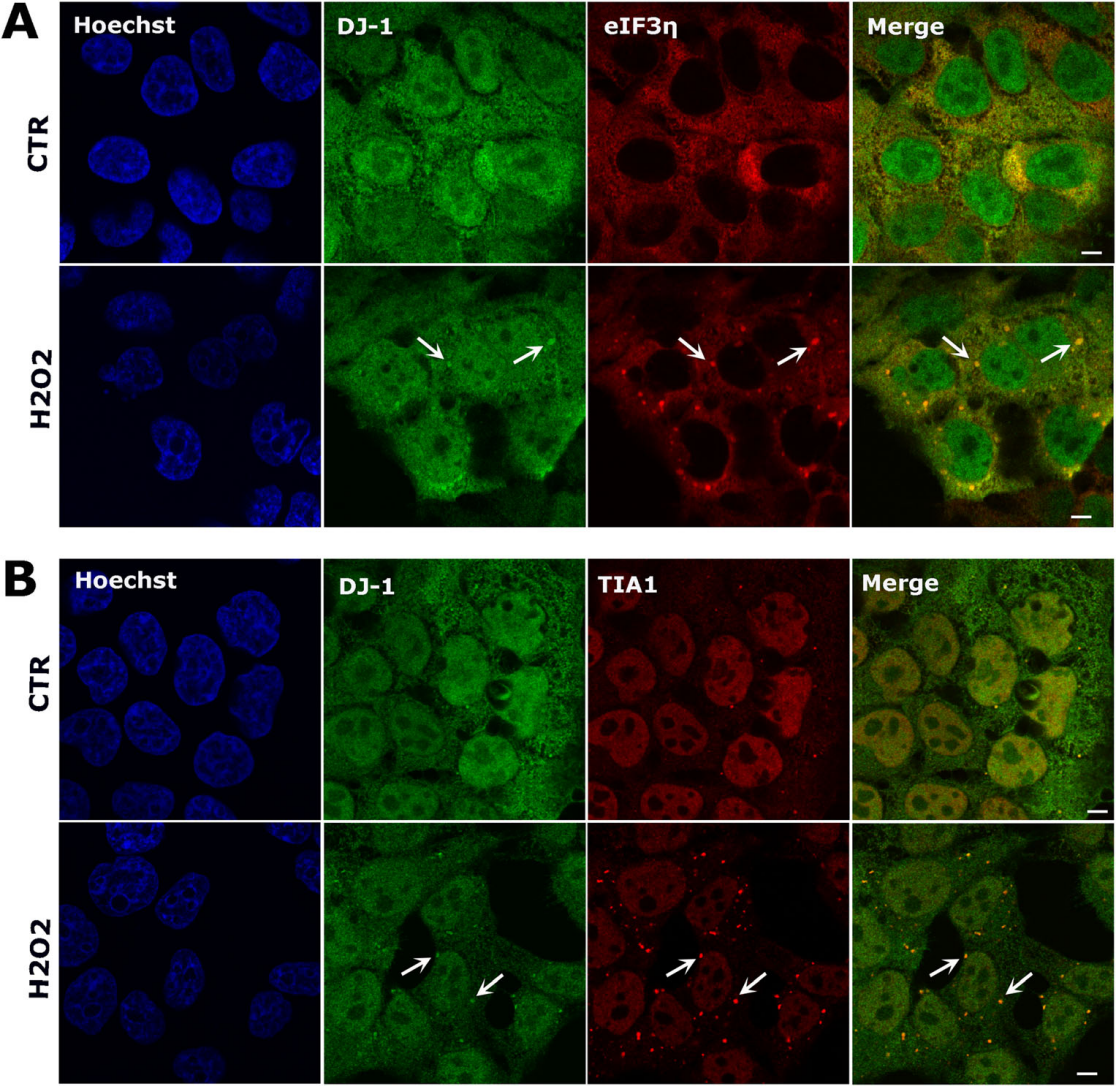
DJ-1 localizes to stress granules after oxidative stress. Confocal images of untreated HEK 293T cells (top row in each panel) compared to cells treated with 1 mM hydrogen peroxide for 2 h (bottom row in each panel). Double immunostaining for DJ-1 and eIF3η (**a**) and DJ-1 and TIA1 (**b**), shows that DJ-1 co-localizes with some stress granules after oxidative stress. Scale bar = 5 μm

We next asked whether DJ-1 was associated with SGs formed in response to other stimuli. We exposed HEK 293T cells to hydrogen peroxide (H_2_0_2_; 1 mM for 2 h) and assessed SG formation via both eIF3η and TIA1 immunolabeling. Both eIF3η (Fig. 2a) and TIA1 (Fig. 2b) staining showed the presence of SGs in the cytoplasm, which were smaller in size and fewer in number in comparison to those forming in response to sorbitol. Double immunolabeling with anti-DJ-1 antibody confirmed the presence of DJ-1 in both eIF3η and TIA1 positive SGs after H_2_O_2_ treatment (Fig. 2a, b). These data suggest that the association of DJ-1 with SGs is a general feature of stress response in HEK 293T cells and is not stimulus dependent.

**Fig. 3:**
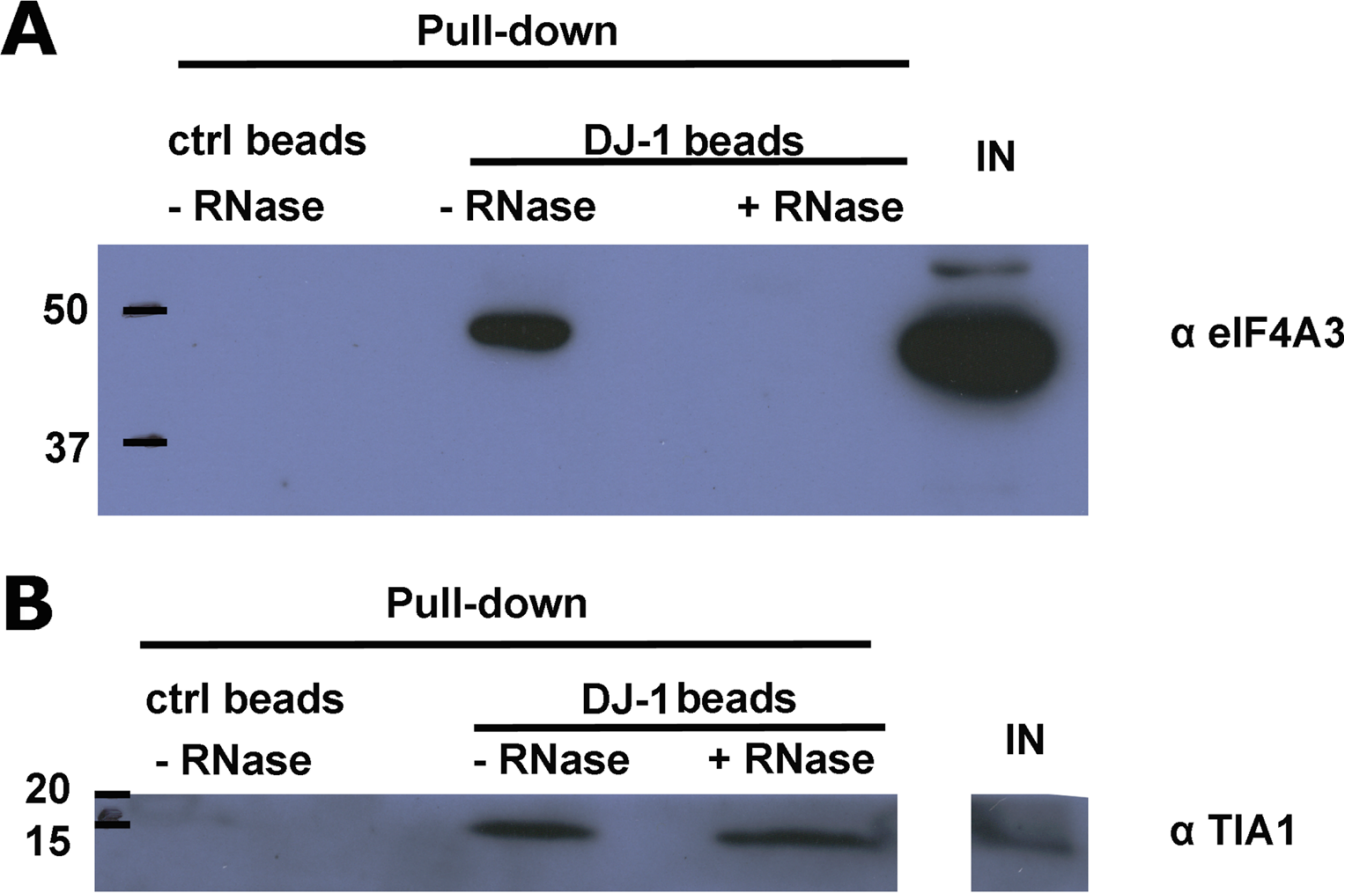
Characterization of the interaction between DJ-1 and associated proteins. The interaction between DJ-1 and eIF4A3 is likely mediated by the binding of both proteins to the same mRNA transcripts, as it clearly disappears after RNAse A treatment (**a**). DJ-1 interaction with the 15 kDa TIA1 protein does not change after RNA depletion (**b**). Images are representative of at least *N* = 3 experiments

SGs are dynamic cytoplasmic structures containing aggregates of mRNA bound to 48S preinitiation factors and RNA binding proteins involved in different aspects of RNA translation or metabolism, as well as proteins that play roles in cellular pathways not directly related to RNA metabolism [39]. Above, we identified two proteins which physically interact with DJ-1 and are known to associate with RNA: eIF4A3 and TIA1, suggesting that these interactions may be mediated by a mutual association with the same mRNA transcript. To investigate this question, we performed coIP assays in the presence or absence of RNase A (Fig. 3) and found that DJ-1 association with eIF4A3 is likely mediated by the binding of both proteins to the same transcripts, as it completely disappears upon RNase treatment (Fig. 3a). Conversely, the interaction of DJ-1 with the 15 kDa TIA1 isoform is not abrogated upon RNase treatment (Fig. 3b), suggesting that protein-protein interactions between TIA1 and DJ-1 occur in the absence of RNA.

**Fig. 4:**
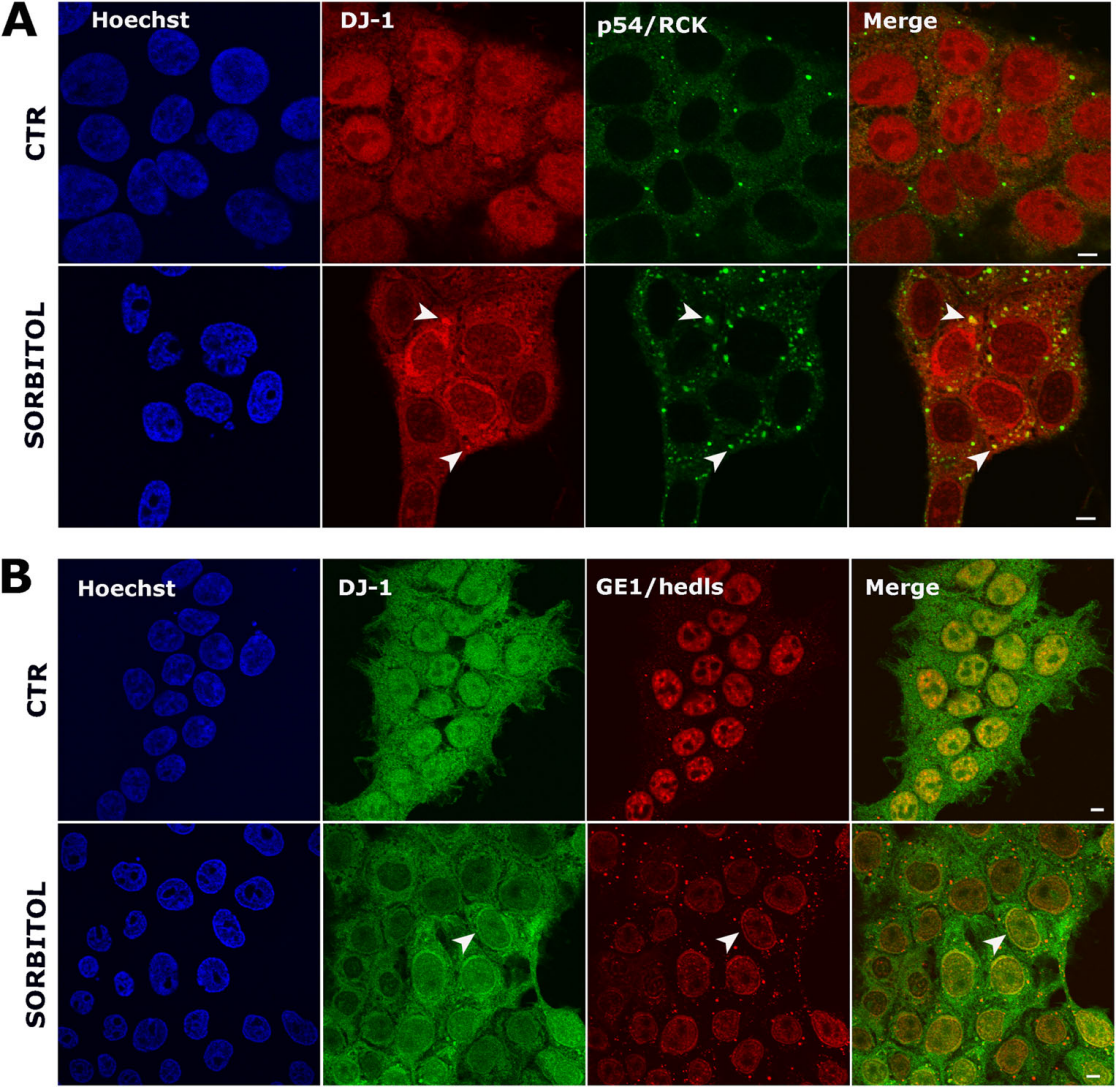
DJ-1 localizes to P-bodies after hyperosmotic stress. Confocal images of untreated HEK 293T cells (top row in each panel) compared to cells treated with 0.4 M sorbitol for 2 h (bottom row in each panel). P-bodies were detected in both untreated and treated conditions. Double immunostaining for DJ-1 and p54/RCK (**a**) shows that DJ-1 co-localizes with some P-bodies after hyperosmotic shock. Double immunostaining for DJ-1 and GE1/Hedls (**b**) clearly indicates co-localization of the two proteins in the perinuclear region. Scale bar = 5 μm

As we previously found that the yeast DJ-1 family member Hsp31 is associated with both SG and PBs—and as the interaction between these structures is highly dynamic—we next explored whether DJ-1 was a component of PBs arising from hyperosmotic shock induced by sorbitol. We employed two PB markers to test for co-localization: p54/RCK and Hedls. As expected, PBs were observed in unstressed cells which increased in both number and size after sorbitol treatment (Fig. 4). No co-localization between DJ-1 and p54 or Hedls was observed in control conditions; however, after hyperosmotic shock, the subcellular localization of DJ-1 changed with a subset co-localizing with p54-labeled PBs (Fig. 4a). DJ-1 and Hedls co-localization in PBs was rarely observed; however, clear overlap of the two fluorescent signals was present in perinuclear regions after sorbitol treatment (Fig. 4b).

### DJ-1 Behaves as a Bona fide SG Component

**Fig. 5:**
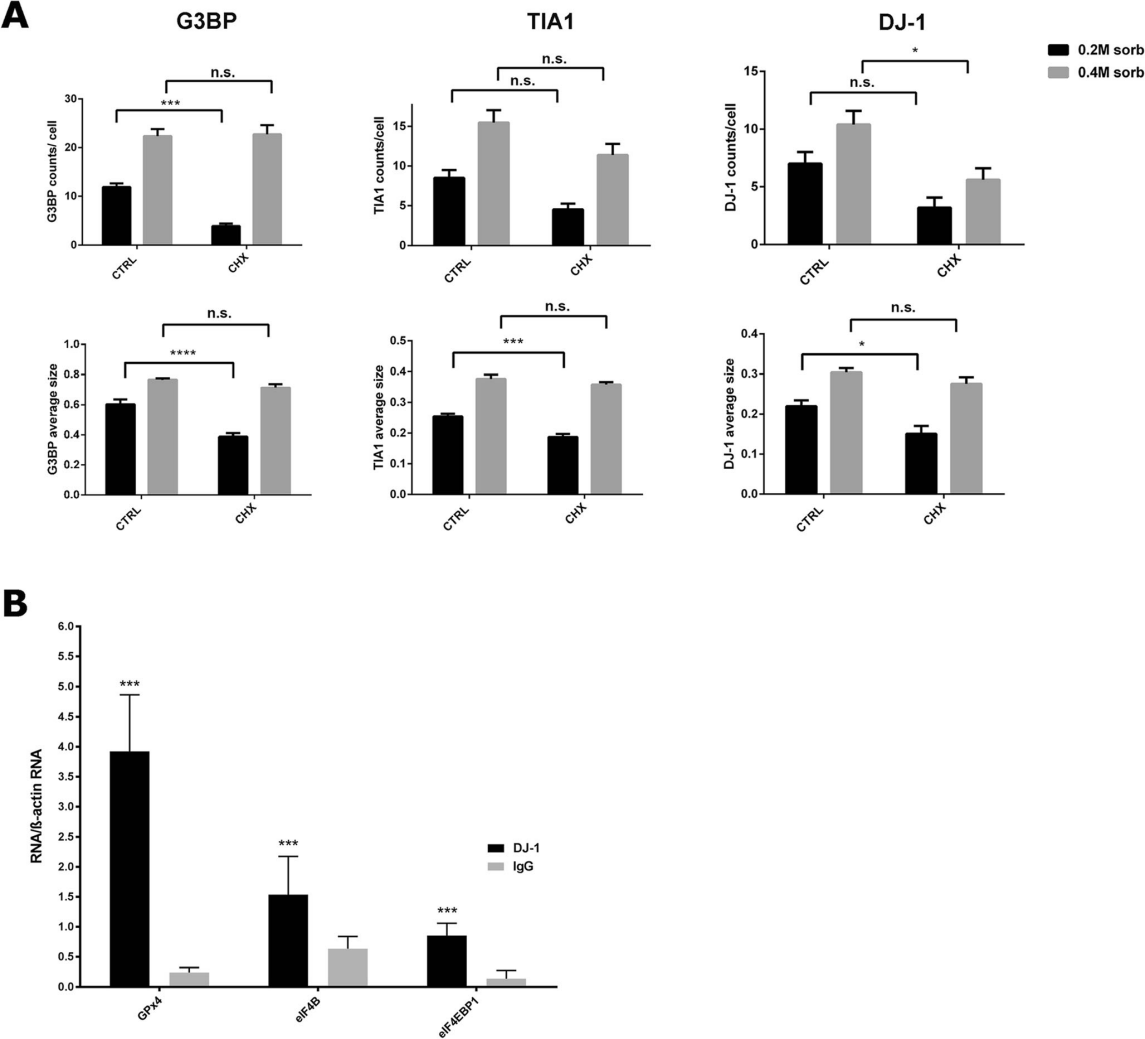
DJ-1 behaves as a bona fide stress granule component and interacts with specific mRNAs. **a** Sensitivity of stress granules to cycloheximide. HEK 293T cells were treated with 0.2 or 0.4 M sorbitol for 30 min and then incubated with cycloheximide for another 30 min in the presence of sorbitol. Cells were fixed and stained with anti-G3BP, anti-TIA1, and anti-DJ-1 antibodies. Cycloheximide causes a reduction in the number and size of G3BP positive stress granules size and a reduction in size of TIA1 positive stress granules as a function of sorbitol concentration. DJ-1 labeling shows a similar reduction after cycloheximide treatment with behaviour comparable to TIA1. ~ 600 cells were counted for each condition from two independent experiments. Data are shown as mean ± SEM. Statistical analysis by two-way ANOVA followed by post hoc analysis with a Tukey’s test; *P < 0.05; **P < 0.01; ***P < 0.001; **** *P* < 0.0001. **b** DJ-1 interacts with specific mRNAs in HEK 293T cells. Cells were lysed and anti-DJ-1 and non-specific IgG antibodies were used to immunoprecipitate RNA bound to DJ-1 or background, respectively. The amount of transcripts associated with each sample was measured by QPCR, and levels of β-actin mRNA were used as a non-specific control. By normalizing to β-actin mRNA levels in each sample, an enrichment of the three transcripts was observed in DJ-1 IP samples compared to IgG IP samples (increase of ~ 16-fold, ~ 2.4-fold, and ~ 6.1-fold, respectively, for *GPx4*, *eIF4B*, and *eIF4EBP1*). Statistical significance was determined by comparing DJ-1 to IgG IP; ***P < 0.001

The RNA and protein components of SGs are highly dynamic and in equilibrium with polysomes [39, 40]. Indeed, it is possible to trap SG components into polysomes by inhibiting translation elongation [41, 42]. Thus, to further confirm that DJ-1 is behaving as a bona fide SG protein, we next explored the effect of cycloheximide (CHX) on stress granule formation. HEK 293T cells were treated with either 0.2 or 0.4 M sorbitol for 30 min before CHX was added to the culture medium for an additional 30 min. Cells were then fixed, labeled with anti-G3BP, anti-TIA1, and anti-DJ-1 antibodies, and the number and size of SG were evaluated as described above (Fig. 5a). Our results strongly support the notion that the DJ-1 associated structures are true SGs, as the CHX effect we observed was a function of sorbitol concentration. Indeed, no CHX effect was detected at 0.4 M for G3BP positive stress granules, while a strong reduction of G3BP positive granules/cell and size was present when cells were treated with 0.2 M sorbitol. This indicates that 0.4 M sorbitol completely blocks translation initiation thereby preventing the CHX treatment effect, which was readily detected with 0.2 M sorbitol, when residual translation was likely still occurring. A similar response to CHX treatment was observed when we used TIA1 as a SG marker, although in this case, there was more of an effect on granule size than on the number of granules per cell. Interestingly, the behavior of DJ-1 positive granules mirrored what we observed with TIA1 positive granules. These data indicate that DJ-1 can likely shift from polysomes to SG as a function of sorbitol concentration, as well as further underscoring that DJ-1 and TIA1 are highly associated within SGs.

### DJ-1 Plays a Role in SG Dynamics

The above evidence of DJ-1 association with SGs and PBs prompted us to explore the nature of this involvement. First, we tested whether DJ-1 could play a role in determining the size or number of SGs/PBs. We employed RNAi to knockdown *PARK7*, which abrogated DJ-1 protein expression 72 h after transfection (Fig. S2G), and subsequently scored SG number and size. Seventy-two hours after transfection, cells were treated with 0.4 M sorbitol for either 2 h (Fig. S2A) or 30 min (Fig. S2B), fixed and labeled with an anti-G3BP (Fig. S2A) or an anti-eIF3η antibody (Fig. S2B). Knockdown of DJ-1 did not have any effect on the number or size of SGs evaluated using these two different markers. As 0.4 M sorbitol provides a relatively strong hyperosmotic shock—and under these conditions translation is likely to be completely blocked—we repeated the experiment in milder conditions using 0.2 M sorbitol for 1 h and we labeled SG with eIF3η (Fig. S2C) or TIA1 antibody (Fig. S2D). Again, no effect of DJ-1 silencing was observed compared to control cells. We further analyzed the number and size of PBs upon DJ-1 knockdown both in control conditions (Fig. S2E) and after 1 h 0.4 M sorbitol treatment (Fig. S2F) using p54 and Hedls as markers. As with the SGs, we did not detect any significant changes upon depletion. Thus, unlike our observations in yeast, we find that although DJ-1 is localized to SGs and PBs upon stress induction, it is not involved in formation of these structures at a gross level.

We next asked whether DJ-1 plays a role in mRNA dynamics within SGs, as mRNAs are core components of SGs/PBs and DJ-1 has been shown to associate with specific mRNA targets in vitro and in vivo [7, 8]. First we verified by RT-PCR the expression of several previously identified DJ-1-associated transcripts in HEK 293T cells (data not shown) and focused our attention on the *GPx4*, *eIF4B*, and *eIF4EBP1* mRNAs due to their relevant cellular roles. The glutathione GPx4 is a key antioxidant protein, while eIF4B protein is a SG component [10] and eIF4EBP-1 regulates the SG localization of eIF4E [43]. DJ-1-mRNA complexes were immunoprecipitated from HEK 293T cell lysates and associated mRNAs were isolated and detected by QPCR. We found that the *GPX4*, *eIF4B*, and *eIF4EBP1* transcripts were significantly enriched in the DJ-1-immunoprecipitated samples as compared to IgG controls (Fig. 5b), confirming the specificity of DJ-1 interaction with these mRNAs in HEK 293T cells.

**Fig. 6:**
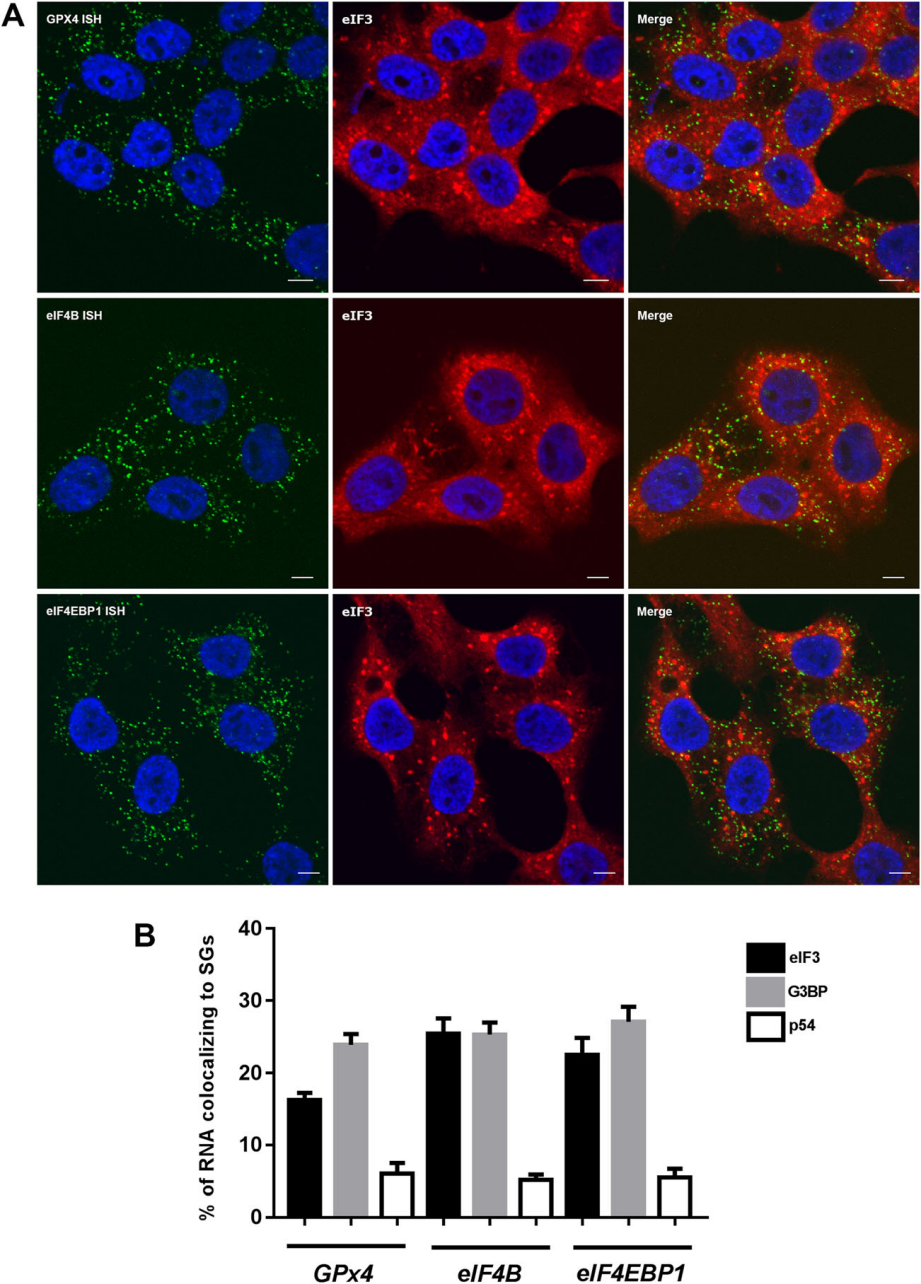
DJ-1 interacting mRNAs localize to stress granules upon induction of stress. **a** Double RNAscope in situ hybridization and eIF3η immunohistochemistry. The merged picture shows co-localization of a subpopulation of mRNAs to eIF3 positive granules. Scale bar = 5 μm. **b** An enrichment of RNA dots representing *GPX4*, *eIF4B*, and *eIF4EBP1* RNA messages localized to stress granule markers (eIF3η and G3BP) was observed in comparison to a P-body marker (p54)

These data further stimulated us to investigate if the DJ-1 interacting transcripts localized to SGs induced by hyperosmotic shock. RNA in situ hybridization (ISH) using the RNAscope Multiplex Assay permitted visualization of individual RNAs as single, small fluorescent dots [44] which were analyzed in combination with immunofluorescence (IF) for SG markers. Our data clearly show that this ISH approach specifically detects the presence of the three candidate mRNAs in HEK 293T cells (Fig. S3). We then used ISH to detect each of the three transcripts in combination with IF with SG markers (eIF3η and G3BP) after 1 h sorbitol treatment. The combination of detecting the mRNAs in the green channel (488 nm) and SGs in the far-red channel (647 nm) provided good contrast (Fig. 6a) in agreement with previous results [45]. Interestingly, we found that ~ 20% of *GPX4* mRNA signal co-localized with the two SG markers (Fig. 6b). Similarly, ~ 25% of eIF4B and eIF4EBP1 co-localized to these markers. Notably, only ~ 5% of the mRNA signals co-localized to the PB control, indicating a significant enrichment of these candidate mRNAs with SGs versus other RNA granules (e.g., PBs). These data suggest that a subset of mRNAs may be targeted to SGs by DJ-1.

### DJ-1 Is Associated with SGs in Neuronal Models of Cellular Toxicity

**Fig. 7:**
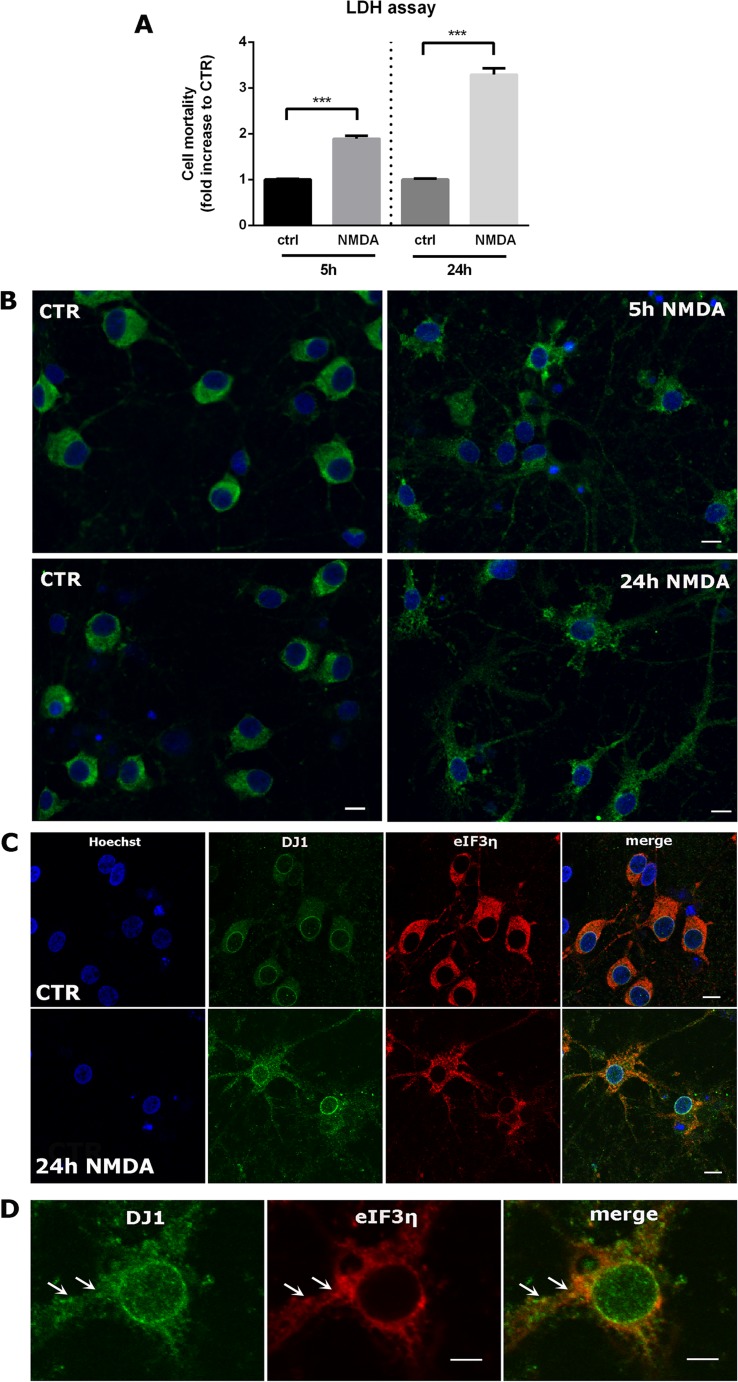
NMDA excitotoxicity induces DJ-1 positive stress granules in primary cortical neurons. **a** Neuronal death assayed by LDH release after 5 and 24 h NMDA exposure. Quantifications were performed in at least four independent experiments. Data are shown as mean ± SEM. **b** Confocal images of primary cortical neurons untreated (left panels) compared to neurons treated with NMDA for 5 or 24 h (right panels). eIF3η immunolabeling indicates that NMDA induces stress granules at both 5 and 24 h treatment. Scale bar = 10 μm. **c** Cortical neurons were treated with NMDA for 24 h, fixed and double immunolabeled for eIF3η and DJ-1. DJ-1 changes its cellular distribution becoming more dotted and distributed in the neuropil after 24 h NMDA treatment. Scale bar = 10 μm. **d** Higher magnification images show co-localization of the two proteins mainly in the perinuclear region. Scale bar = 5 μm. Images are representative of at least *N* = 3 experiments

We also considered the role of DJ-1 and SGs in cellular toxicity models relevant for neurodegenerative disease. In this regard, we first employed a primary rat neuronal model of excitotoxicity. In the CNS, excessive *N*-methyl-d-aspartate (NMDA) receptor activation leads to excitotoxicity, an important mechanism involved in cell death in many acute and degenerative neurological disorders [46]. In PD, in particular, the loss of dopaminergic neurons in the *substantia nigra pars compacta* results in an excessive glutamatergic input into several areas of the basal ganglia [47] and NMDA receptors present on dopaminergic neurons are the targets for PD therapeutic drugs [48, 49]. As we have previously extensively characterized a model of NMDA excitotoxicity in rat primary cortical neurons (excitotoxicity induced at 12 DIV by treatment with 100 μM NMDA [50, 51]), we employed this model to ascertain whether this treatment induces SGs in primary neurons, which is likely relevant to other neuronal types, including dopaminergic neurons. We first looked for the presence of SGs in NMDA-treated neurons versus control cells. Notably, we found that NMDA receptor overactivation induces formation of SGs in primary cortical neurons, as indicated by both eIF3η and TIA1 labeling (Fig. 7b, data not shown), indicating that excitotoxicity causes a reorganization of the RNA machinery in neuronal cells. Indeed, while in control conditions eIF3η signal is mainly cytoplasmic, after 5 h NMDA treatment, it becomes punctate and spreads into the neuropil, with this change in protein localization being even more evident after 24-h treatment (Fig. 7b). Similar results were obtained using TIA1 as a SG marker (data not shown). We investigated DJ-1 in this model and found that after 24 h of NMDA treatment, DJ-1 changes its cellular localization in a similar way to that observed for eIF3η, with a fluorescent signal predominantly localized to axons and dendrites and to the perinuclear region (Fig. 7c), where we observed co-localization with the eIF3η signal.

**Fig. 8:**
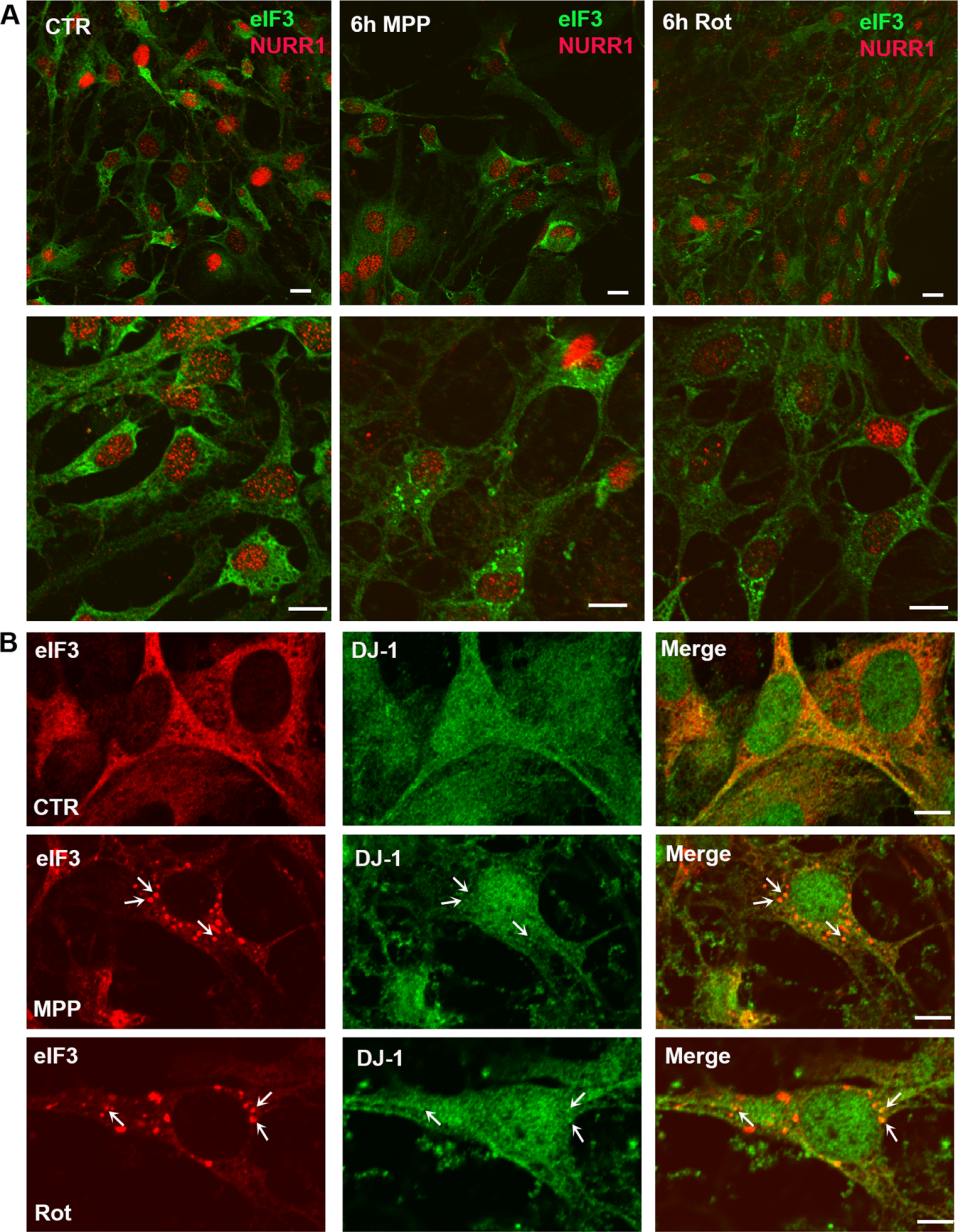
Parkinsonian neurotoxins induce DJ-1 positive stress granules in dopaminergic cell cultures. **a** Confocal images of control dopaminergic cultures and dopaminergic cultures treated with 20 μM MPP+ or 50 nM rotenone for 6 h (upper panel =× 20 magnification, lower panel =× 40 magnification). Double immunostaining for eIF3 and NURR1 shows the presence of SGs in in cells with a low level of NURR1. (Scale bar = 10 μm). **b** DJ-1 co-localizes to SGs arising from MPP+ or rotenone treatment in dopaminergic cultures (6 h, Rotenone 50 nM or MPP+ 20 μM). Scale bar = 5 μm

Finally, we considered whether mitochondrial toxins causing the selective degeneration of *substantia nigra* dopaminergic neurons in PD could induce SGs associated with DJ-1. We derived homogeneous cultures of dopaminergic neurons from mouse ES cells expressing Lmx1a under control of the Nestin enhancer (NesE-Lmx1a) [22, 23] and treated these cultures with either 20 μM MPP^+^ or 50 nM rotenone for 3, 6, 12, or 24 h. The presence of SGs was evaluated using immunofluorescence with eIF3η signal as a marker for these RNA granules. Strikingly, we observed that both of these mitochondrial toxins induced formation of SGs at the early time points of 3 and 6 h (Fig. 8a), while at the later time points of 12 and 24 h, these RNA granules were no longer visible (data not shown). In control cultures, SG formation was never observed. Labeling of these neuronal cultures with the dopaminergic neuronal marker Nurr1 after these toxic insults found that SGs were only present in cells with diminished expression of this marker, while neurons that did not show SG formation expressed normal levels of Nurr1 (Fig. 8a). Notably, while the majority of cells (~ 70%) in culture were tyrosine hydroxylase (TH)-positive, as expected, SG-containing cells never showed TH signal (Fig. S4). Double immunolabeling for DJ-1 and eIF3η indicated that DJ-1 co-localized to the SGs resulting from MPP+ and rotenone (Fig. 8b) treatment. In total, these data suggest that alterations in the RNA machinery may arise in models of neuronal toxicity, and that DJ-1 may play a role in this process.

## Discussion

Here, we present novel observations that DJ-1 is associated with mRNP granules, suggesting a potential role for DJ-1 in modulation of the RNA machinery which may be important for its pathogenic role in PD and other neurodegenerative disorders. Specifically, we identified several SG components and related proteins (eIF4A3, hnRNP family proteins, and 40S ribosomal proteins) as DJ-1 interacting proteins by mass spectrometry, with subsequent coIP experiments validating eIF4A3 and hnRNPM as DJ-1 interactors. eIF4A3 is a member of the eIF4A family of DEAD-box RNA helicases, three of which have been described in vertebrates (eIF4A1, eIF4A2, eIF4A3). eIF4A3 is a core component of the exon junction complex (EJC) [26], resides in the nucleus, and may serve as an RNA clamp escorting spliced mRNAs from the nucleus to various cellular compartments [52], while eIF4A1 and eIF4A2 are cytoplasmic proteins whose helicase activity is stimulated by their binding partners eIF4G and eIF4H. Notably, eIF4A family members are core components of SGs [10, 53] and eIF4A3 has recently been described as a member of the SG proteome [32]. We found that the interaction between DJ-1 and eIF4A3 is likely dependent upon the presence of mRNA transcripts associated with both proteins and that upon sorbitol treatment DJ-1 and eIF4A3 strongly co-localized to the perinuclear region (data not shown). Therefore, it is possible that DJ-1 works in concert with eIF4A3 to target spliced mRNAs from the nucleus to SGs in the cytoplasm during stress. Remarkably, eIF4A3—as a component of the EJC—is involved in the pioneer round of translation, which is critical for RNA quality control [54]. hnRNPs are also RNA binding proteins that have been linked to several aspects of RNA metabolism (for a review see [55]). hnRNPA1 [27, 28], hnRNPA2 [29], and hnRNPK [30] have all been described as SG components. Notably, *Caenorhabditis elegans* SUP-46, an RNA binding protein with homology to human hnRNPM, was found to localize to SGs after heat stress [56]—further suggesting that hnRNPM may also be a SG protein.

We also observed that DJ-1 strongly interacts with the 15 kDa SG-associated form of TIA1 comprising a C-terminal region arising from proteolytic cleavage [57], a well-described SG marker. Interestingly, this association is RNA-independent, suggesting that DJ-1 interacts with TIA1 by a different mechanism than with eIF4A3. Furthermore, upon SG induction by two different stimuli (osmotic shock and oxidative stress), we clearly observed co-localization of DJ-1 and SGs labeled with TIA1, G3BP, and eIF3 in HEK 293T cells, and co-localization of DJ-1 with TIA1 and tau in SH-SY5Y cells after sorbitol treatment (Fig. S5, Fig. 1d). To further confirm a biological role of DJ-1 in SGs, during stress conditions we observed partial co-localization of DJ-1 with PBs, highly dynamic RNA granules which can exchange components with SGs [11, 58]. Finally, we found that DJ-1 likely shifts from polysomes to SGs as a function of sorbitol concentration, further supporting that DJ-1 is a bona fide SG component. These data conclusively show for the first time, to our knowledge, the association of DJ-1 with SGs in mammalian cells, leading us to explore its functional relevance.

Notably, our results herein suggest that DJ-1 does not play a role in size or number of SGs under the conditions tested. As DJ-1 has previously been found to specifically bind a subset of mRNAs [7, 8], we thus next asked whether DJ-1 targets a subset of mRNAs to SGs. Supporting this hypothesis, we performed ISH with *GPx4*, *eIF4B*, and *eIF4EBP1* mRNAs as candidate markers, and found an enrichment of these messages co-localized to SGs in comparison to PBs, which served as control RNA granules. These data—combined with past work showing that DJ-1/mRNA interactions are abrogated under oxidative stress conditions [7]—suggest that DJ-1 may play a role in regulating translation of these messages during stress.

DJ-1 has recently been shown to deglycate methylglyoxal- and glyoxal-glycated Cys, Arg, and Lys protein residues [59] as well as methylglyoxal- and glyoxal-glycated nucleotides and nucleic acids [60]. Strikingly, O-Glc-NAc glycosylation of proteins enhances SG formation [61] and advanced glycation end products induce SG assembly in human chondrocytes [62]. Our recent work has highlighted the likely importance of glycation in the pathogenesis of PD and Huntington’s disease [63, 64]. Thus, based upon these reports, it is possible that DJ-1 deglycates both proteins and nucleic acids within SGs.

We next explored DJ-1-associated SGs in models of neurodegeneration. Employing a well-established model of NMDA excitotoxicity in primary cortical neurons [50], we observed redistribution of eIF3 and TIA1 proteins from the cytoplasm to the neuropil of cortical neurons and formation of SGs. As described for HEK 293T cells, DJ-1 co-localizes to eIF3η-labeled SGs. Complementing these novel findings, the presence of SGs directly correlated with NMDA-mediated toxicity in the culture as determined by LDH (data not shown). To our knowledge, this indicates for the first time that overactivation of NMDA receptors affects RNA metabolism, thus adding a new component to the complex picture of excitotoxicty and related molecular pathways [46]. Similar analyses with MPP+ and rotenone found that these neurotoxins promote the formation of DJ-1-associated SGs in dopaminergic neuronal cultures at early timepoints (3 and 6 h), which dissipate at longer treatment times (24 and 48 h). Notably, the SG-containing cells in these dopaminergic neuronal cultures lack TH-staining and present with low levels of NURR1 expression. Since MPP+ is selectively taken up by dopaminergic neurons [65], cells showing SG formation might initially have expressed TH and Nurr1. This raises the possibility that SG formation occurs either before or during the decline of Nurr1 and TH expression, ultimately causing neuronal death. While the functional relationship among these observations is not clear, these novel insights suggest that DJ-1-associated SGs may be mechanistically linked to the parkinsonian phenotypes generated by these toxins.

Taken together, our results suggest that DJ-1 may be involved in the process of RNA triage in cells upon induction of stress, which may be relevant to pathogenic mechanisms underlying PD, and other neurodegenerative disorders which exhibit DJ-1-related pathology. As DJ-1 has been linked to a number of cellular processes, a role in RNA metabolism in which specific mRNA populations are regulated by DJ-1 is feasible. How, and if, such mechanisms contribute to disease processes is unclear, but the link with PD-related toxins is indicative. Nonetheless, it is now critical to follow on from these studies by identifying and characterizing the mRNA populations targeted by DJ-1 in the context of SG formation and neurodegeneration, and understanding the downstream effects of these interactions. Ultimately, such analyses will further clarify how loss of DJ-1 function leads to PD, providing important insight into the molecular pathogenesis of this disorder.

## Electronic supplementary material

ESM 1 (PDF 536 kb)

ESM 2 (XLSX 71 kb)
