# Supplementary material for: The Parkinson’s Disease-Linked Protein DJ-1 Associates with Cytoplasmic mRNP Granules During Stress and Neurodegeneration
Source: Mol Neurobiol. 2018 Apr 19;56(1):61–77. doi: 10.1007/s12035-018-1084-y (PMC6334738; doi:10.1007/s12035-018-1084-y)
Supplement: Supplementary file 1 — (PDF 536 kb) [file 12035_2018_1084_MOESM1_ESM.pdf]

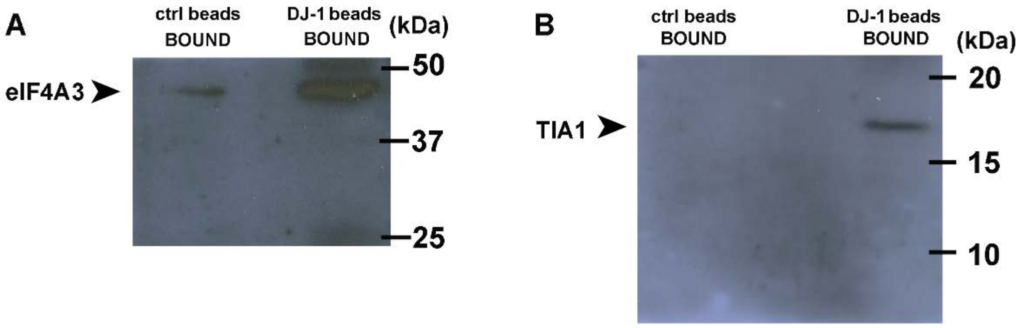

Supplementary Figure 1. eIF4A3 and TIA1 interact with DJ-1 in HEK 293T cells. DJ-1 interacting proteins were immunoprecipitated from HEK 293T lysates (control condition, A; sorbitol treatment, B) with antibodies anti-DJ-1 and the immunoprecipitates were probed by immunoblotting with antibodies anti-eIF4A3 (A) or TIA1 (B). Images are representative of at least three experiments.

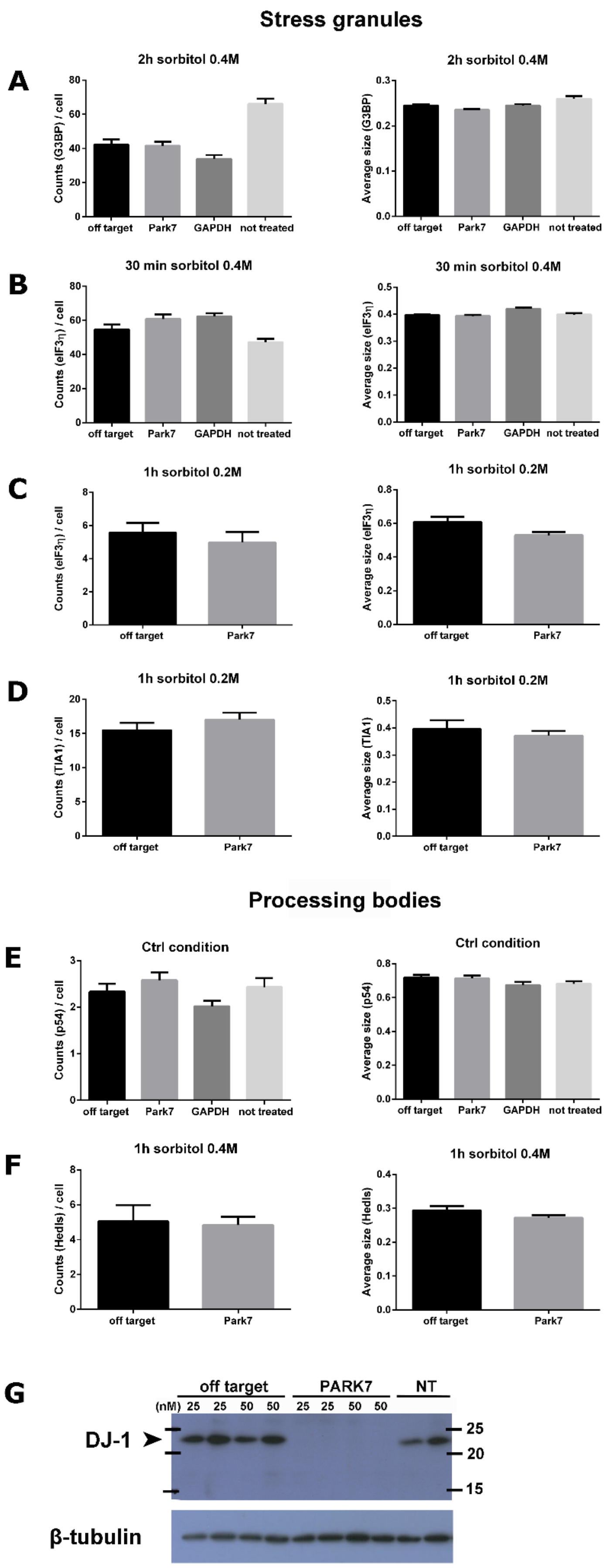
Stress granules
A
2h sorbitol 0.4M
Counts (G3BP) / cell
Average size (G3BP)
off target
Park7
GAPDH
not treated
B
30 min sorbitol 0.4M
Counts (eIF3η) / cell
Average size (eIF3η)
C
1h sorbitol 0.2M
Counts (eIF3η) / cell
Average size (eIF3η)
D
1h sorbitol 0.2M
Counts (TIA1) / cell
Average size (TIA1)
Processing bodies
E
Ctrl condition
Counts (p54) / cell
Average size (p54)
F
1h sorbitol 0.4M
Counts (Hedls) / cell
Average size (Hedls)
G
off target
PARK7
NT
(nM) 25 25 50 50 25 25 50 50
DJ-1
25
20
15
β-tubulin

Supplementary Figure 2. DJ-1 depletion does not affect assembly of stress granules or P-bodies. HEK 293T cells were transfected with 25 nM DJ-1 siRNA for 72 h and treated with 0.4 M sorbitol for 2 h (A), 30 min (B) or 0.2 M sorbitol for 1 h (C and D) in fresh medium. The cells were fixed, and SGs were visualized by labeling with anti-G3BP (A), anti-eIF3η (B and C), or anti-TIA1 (D). The histogram represents the number of SG labelled with the indicated marker (right) as well as SG average size (left). ~1000 cells were counted for each condition from three independent in (A) and (B), and 700 cells for each condition were counted in (C) and (D) from two independent experiments. Data are shown as mean ± SEM. No significant effect was observed when DJ-1 was depleted. The same results were obtained for P-bodies in control conditions (E) or after 0.4M sorbitol treatment for 1 h (F) using p54/RCK (E) and Hedls (F) as markers. At least 1800 cells were counted for each condition from three independent in (E) and 700 cells for each condition were counted in (F) from two independent experiments. Data are shown as mean ± S.EM. (G): Silencing of DJ-1 assessed by immunoblot analysis 72 h after transfection. NT, not transfected cells.

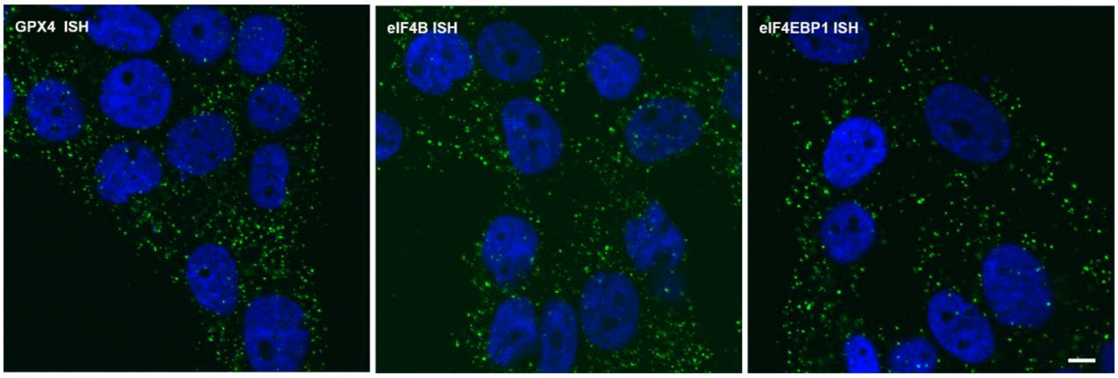

Supplementary Figure 3. RNAscope *in situ* hybridization of candidate mRNAs. Representative images of RNAscope ISH (green) using mRNA probes for *GPx4*, *eIF4B* and *eIF4EBP1* in HEK 293T cells. Scale bar = 5 µm.

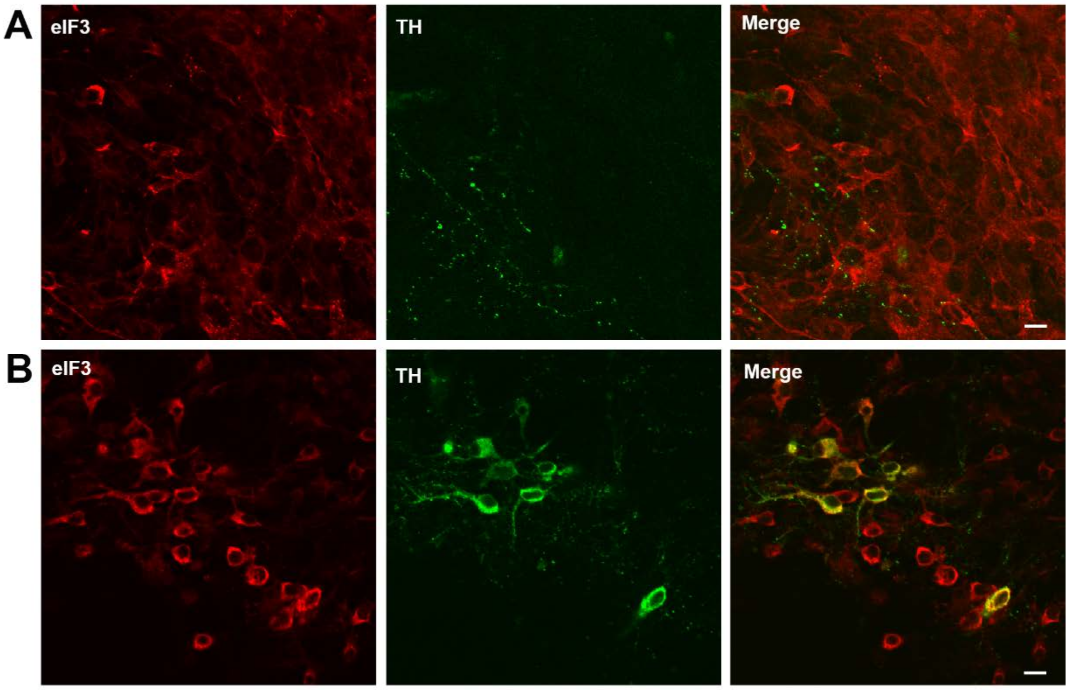

Supplementary Figure 4. SG containing cells do not show TH positive labelling after neurotoxic treatment. Dopaminergic cell cultures were treated with Rotenone 50 nM for 6 h. Double immunostaining for eIF3 and TH clearly indicate the presence of SGs in treated cultures in cells with no visible TH staining (A), while a strong TH signal was observed in cells lacking SGs (B). Scale bar = 10 µm.

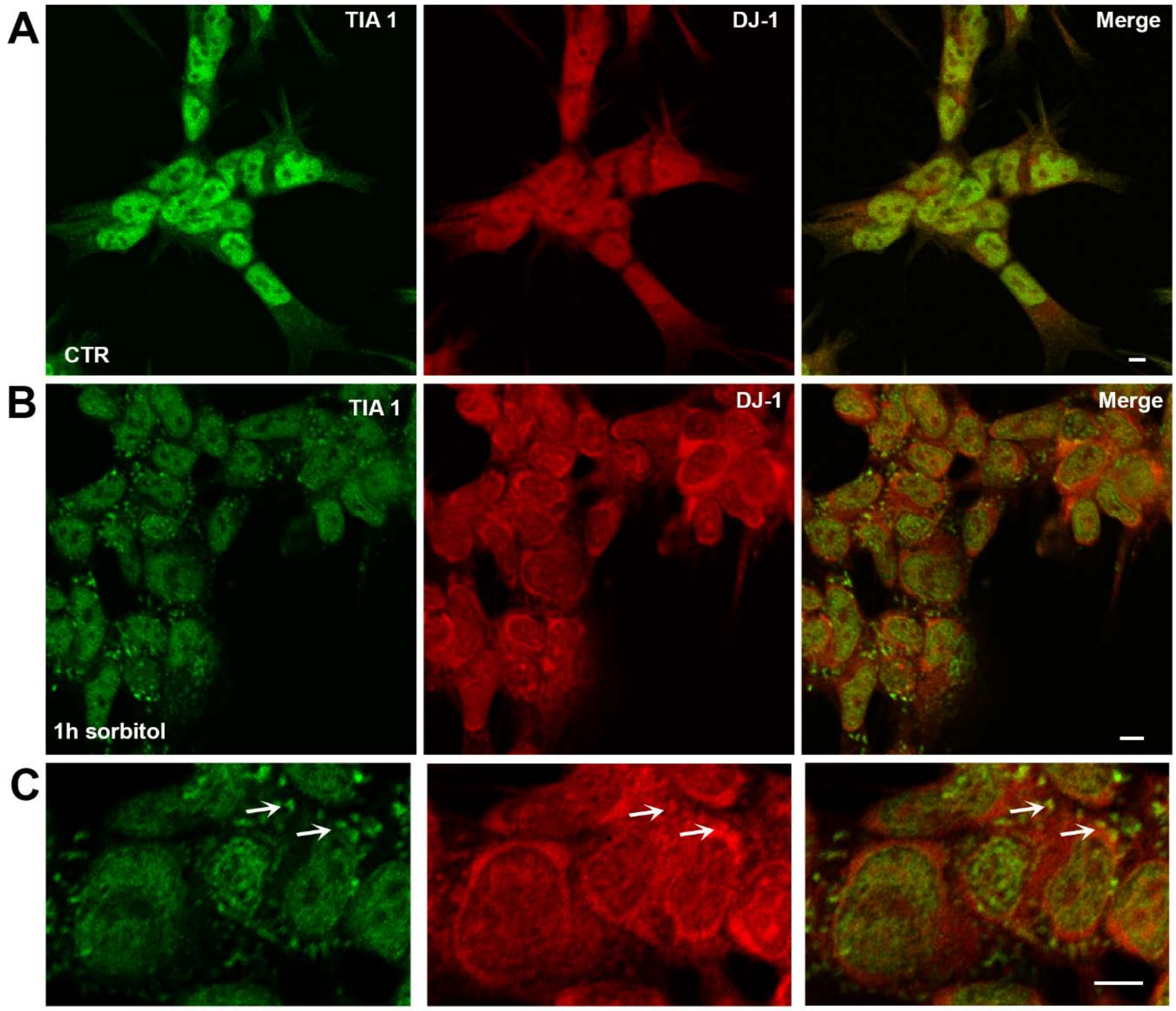

Supplementary Figure 5. DJ-1 localizes to stress granules after hyperosmotic stress in neuroblastoma cells.
Confocal images of untreated SH-SY5Y cells (A) compared to cells treated with 0.4 M sorbitol for 1h (B), after double immunostaining for TIA1 and DJ-1. Sorbitol induces TIA1 positive stress granules and DJ-1 changes its cellular distribution becoming more dotted and perinuclear as already shown in HEK 293T cells. Double immunostaining indicates co-localization of DJ-1 with some stress granules (C). Images are representative of at least N = 3 experiments. Scale bar = 5 µm.

Supplementary Table 1: Mass spectrometry results. DJ-1 interacting proteins pulled down with endogenous or overexpressed DJ-1 in HEK 293T cells.
